# ACLY facilitates alanine flux in the livers of db/db mice: a hyperpolarized [1-13C]pyruvate MRS study

**DOI:** 10.3389/fendo.2025.1663958

**Published:** 2025-10-27

**Authors:** Young-Suk Choi, Jae Eun Song, Seo-Hyun Lim, Ho-Taek Song

**Affiliations:** ^1^ Department of Radiology and Research Institute of Radiological Science, Yonsei University College of Medicine, Seoul, Republic of Korea; ^2^ Department of Transdisciplinary Medicine, Seoul National University Hospital, Seoul, Republic of Korea

**Keywords:** ATP citrate lyase, gluconeogenesis, alanine aminotransferase, hyperpolarized 13C MRS, diabetes, NAFLD

## Abstract

**Introduction:**

Non-alcoholic fatty liver disease (NAFLD) and type 2 diabetes mellitus (T2DM) feature paradoxical increases in both gluconeogenesis and lipogenesis. ATP citrate lyase (ACLY) supports both processes by generating cytosolic acetyl-CoA and oxaloacetate from citrate. While ACLY’s role in lipogenesis is well established, its involvement in amino acid–driven gluconeogenesis remains unclear.

**Methods:**

Using hyperpolarized [1-^13^C]pyruvate magnetic resonance spectroscopy (MRS), we observed [1-^13^C]alanine labeling in the livers of db/db mice. To test the effect of ACLY inhibition, mice were treated with BMS-303141, and blood glucose responses, hyperpolarized alanine labeling, and aminotransferase activity were evaluated. Western blotting was performed to assess ACLY phosphorylation.

**Results:**

Hyperpolarized alanine labeling was markedly elevated in db/db livers, reflecting enhanced transamination capacity. Pharmacologic ACLY inhibition attenuated alanine- and glutamine-induced hyperglycemia and normalized alanine labeling within 2–4 h, without altering aminotransferase gene expression. These in vivo changes correlated with increased hepatic ACLY phosphorylation and ex vivo ALT assay results.

**Discussion:**

Together, these findings support a model in which ACLY facilitates amino acid–driven gluconeogenesis through metabolic control of ALT-mediated transamination, consistent with increased pyruvate–alanine exchange. Hyperpolarized [1-^13^C]pyruvate MRS thereby provides a sensitive, translational readout of dynamic hepatic metabolism relevant to NAFLD and T2DM.

## Graphical Abstract

ACLY promotes ALT1-mediated alanine flux and gluconeogenesis in the diabetic liver. Its inhibition reverses both amino-acid and lipid-driven metabolic dysregulation, as visualized by [1-^13^C]pyruvate MRS.

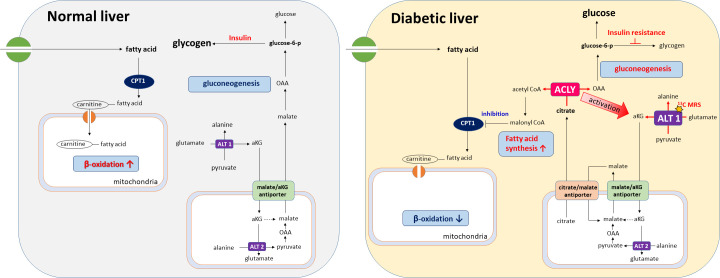

## 1 Introduction

Non-alcoholic fatty liver disease (NAFLD) is a global health problem that affects 25% of the world’s population (1). A hallmark of both NAFLD and type 2 diabetes mellitus (T2DM) is a paradoxical increase in endogenous glucose labeling alongside *de novo* lipogenesis in the liver (2, 3). Several hypotheses have been proposed to explain this metabolic paradox. One posits that preferential activation of mammalian target of rapamycin complex 1 (mTORC1) via AKT, with insufficient inhibition of Forkhead box protein O1 (FoxO1), results in elevated lipogenesis while failing to suppress gluconeogenesis (4, 5). Another hypothesis suggests that hyperinsulinemia-induced hepatic lipogenesis may be a key driver of this metabolic dysregulation (6). However, this view remains controversial, as some studies have shown that hepatic lipid synthesis may be minimal following glucose ingestion (7). Thus, the molecular mechanisms underpinning the simultaneous activation of lipogenesis and gluconeogenesis in NAFLD and T2DM remain incompletely understood.

ATP citrate lyase (ACLY) is a cytosolic enzyme that converts mitochondrial-derived citrate into acetyl-CoA and oxaloacetate (OAA), precursors for lipogenesis and gluconeogenesis, respectively. Acetyl-CoA fuels *de novo* lipid synthesis, while OAA contributes to gluconeogenesis via phosphoenolpyruvate carboxykinase (PEPCK) (8, 9). ACLY functions as a metabolic hub that links carbohydrate, lipid, and energy metabolism through its dual role. Inhibition of ACLY has been shown to alleviate hepatic steatosis and improve glucose control in animal models of metabolic syndrome (10–12). While ACLY’s involvement in lipogenesis is well established, its functional role in amino acid–driven gluconeogenesis remains less well defined.

Hyperpolarized (HP) ^13^C magnetic resonance spectroscopy (MRS) enables real-time, noninvasive monitoring of metabolic fluxes *in vivo* by dramatically enhancing the signal of ^13^C-labeled substrates (13, 14). When administered as [1-^13^C]pyruvate, this tracer can be metabolized to lactate, alanine, or bicarbonate through lactate dehydrogenase (LDH), alanine aminotransferase (ALT), or pyruvate dehydrogenase (PDH), respectively. Recent work has demonstrated that HP [1-^13^C]pyruvate MRS can effectively detect altered liver metabolism in models of NAFLD and T2DM (15–17).

In this study, we used HP [1-^13^C]pyruvate magnetic resonance spectroscopy (MRS) to examine hepatic metabolic fluxes in db/db mice. During this process, we observed a striking increase in the [1-^13^C]alanine signal, indicating enhanced ALT-mediated transamination in the diabetic liver. This unexpected finding led us to hypothesize that ACLY, a key enzyme linking citrate metabolism to both gluconeogenesis and lipogenesis, may play a functional role in amino acid–driven gluconeogenesis. To test this, we investigated the effect of ACLY inhibition on alanine/glutamine-induced hyperglycemia and hepatic alanine flux. Our study further aimed to determine whether such metabolic regulation occurs through transcriptional control or via post-translational modulation of enzymatic activity.

## 2 Methods

### 2.1 Animal procedures

All animal procedures were approved by the Institutional Animal Care and Use Committee (IACUC) of Yonsei University (YLARC; No. 2019-0219). Male C57BLKS/J-db/db mice and age-matched C57BLKS/J-m+/db control mice (Japan SLC, Shizuoka, Japan) were housed under standard conditions (23 °C, 12 h light/dark cycle) with ad libitum access to food and water unless otherwise specified. In our colony, a subset of db/db and db/+ littermates exhibited congenital kidney abnormalities (e.g., hypoplastic or unilateral kidney). Because such defects may confound systemic metabolism, these animals were excluded from the final analysis according to pre-specified exclusion criteria. An overview of the experimental timeline is provided in **Supplementary Figure S1**.

#### 2.1.1 Hyperpolarized [1−^13^C]pyruvate MR spectroscopy

To assess hepatic alanine flux, five db/db and five control mice were subjected to overnight fasting (~20 h) and scanned using HP [1−^13^C]pyruvate MRS at 14–16 weeks of age. Mice with congenital kidney abnormalities were excluded from the analysis, resulting in a final sample size of n=4 per group. To evaluate the acute effect of ACLY inhibition, mice were treated with 2.5 mg/kg BMS-303141 (MCE, USA) via oral gavage after fasting. Follow-up HP [1−^13^C] MRS was performed 2–4 h post-treatment, within the effective window based on the reported 2.1-hour half-life of BMS-303141 at 20–22 weeks (11). Mice were then maintained without further treatment for 6–8 weeks prior to tissue harvesting at 28 weeks, following overnight fasting.

#### 2.1.2 Blood glucose and amino acid tolerance tests

To assess the metabolic effects of ACLY inhibition, db/db mice (18 weeks old) received a single oral dose of 2.5 mg/kg BMS-303141 (prepared at 25 mg/mL in DMSO, diluted in corn oil to 0.625–1.25 mg/mL) after a 20-h fast. Blood glucose was measured using a GlucoDr.Top (Allmedicus, Korea). For amino acid tolerance tests, mice were fasted for 20 h, then injected intraperitoneally with 1 g/kg L-alanine or L-glutamine (Sigma-Aldrich, USA), with or without BMS-303141 pretreatment 2 h prior to injection.

### 2.2 Hyperpolarized ^13^C magnetic resonance spectroscopy

HP [1−^13^C]pyruvate MRS was performed on a 9.4T MRI scanner (Bruker BioSpin MRI GmbH, Ettlingen, Germany) using a 20-mm ^1^H/^13^C dual-tuned coil. [1−^13^C]pyruvic acid (26.7 mg, Cambridge Isotope, Tewksbury, MA, USA), mixed with 15 mM trityl radical OX-063 (Oxford Instruments, Oxford, UK) and 0.75 mM gadoterate meglumine (Dotarem^®^; Guerbet, Villepinte, France), was hyperpolarized in a HyperSense^®^ DNP system (Oxford Instruments, Oxford, UK). Dynamic slice-selective ^13^C-FID data (10°flip, 1 s time resolution, 2 min total) were acquired immediately upon pyruvate injection (~350 µL of 79 mM pyruvate). The apparent conversion rate constants *KpA* (pyruvate to alanine) and *KpL* (pyruvate to lactate) were estimated using a two-site exchange model (18).

### 2.3 Tissue sample preparation

Liver tissues were obtained from the same cohort of mice used for the HP [1−^13^C]pyruvate MRS experiments. After being maintained under the same experimental conditions until 28 weeks of age, mice were euthanized via CO_2_ inhalation after overnight fasting. Liver tissues were immediately frozen in liquid nitrogen and stored at −80°C for subsequent biochemical analyses.

### 2.4 Assessment of lactate and alanine level

Liver tissue was homogenized in NP-40 buffer (50 mM Tris-HCl, pH 7.5, 150 mM NaCl, 5 mM EDTA, and 1% NP-40). The lysate concentration was measured using a BCA protein kit (Thermo Scientific, IL, USA). The lactate concentration was measured using a lactate colorimetric assay kit (K607, Biovision, CA, USA), and the alanine concentration was measured using an alanine colorimetric assay kit (K652, Biovision, CA, USA) according to the manufacturer’s instructions and calculated in 40 μg of lysate.

### 2.5 Assessment of ALT and AST activity assay

Liver tissues were homogenized with the assay buffer and analyzed using commercial colorimetric assay kits for alanine aminotransferase activity (ALT; K752, Biovision, CA, USA) and aspartate aminotransferase activity (AST; K753, Biovision, CA, USA). The lysate protein concentration was measured using a BCA protein kit. ALT and AST enzyme activity assays were performed to the manufacturer’s instructions. These assays reflect apparent enzyme activity under ex vivo assay conditions, rather than direct *in vivo* enzymatic activity.

### 2.6 Western blot

Proteins were isolated using ice-cold RIPA lysis and extract buffer (Thermo Fisher Scientific, Waltham, MA, USA) containing phosphatase inhibitors (5 mM β-glycerophosphoric acid, 10 mM NaF, and 1 mM Na3VO4), 1 mM PMSF, and a protease inhibitor cocktail (Sigma Aldrich, St. Louis, MO, USA). Homogenate containing 15 µg of protein was subjected to 10% SDS-PAGE under reducing conditions. The proteins were transferred to PVDF membranes in transfer buffer at 30 V for 16 h at 4°C. Western blots were subsequently incubated for two hours in 5% skim milk at room temperature and then incubated overnight with a 1:1,000 dilution of anti-phospho–ATP–citrate lyase (Ser455) (4331; Cell Signaling Technology, Danvers, MA, USA), ATP-citrate lyase (4332, Cell Signaling Technology, MA, USA), and anti-β-actin (sc-47778; Santa Cruz Biotechnology, Dallas, TX, USA), washed twice with Tween 20/Tris-buffered saline (TTBS), and incubated with a 1:3,000 dilution of horseradish peroxidase-conjugated secondary antibody for two hours at room temperature. After washing three times with TTBS, the blots were developed using WEST-SAVE Up luminol-based ECL reagent (ABfrontier, Seoul, Korea). The membranes were analyzed using the ImageJ software (National Institutes of Health, Bethesda, MD, USA).

### 2.7 Statistical analysis

All data are presented as mean ± SEM. Time−course data from tolerance tests were analyzed by two−way repeated-measures ANOVA (factors: group × time), followed by Tukey’s *post hoc* tests with adjusted *p*-values. For HP [1−13C]pyruvate MRS, apparent conversion rate constants (*KpA and KpL*) were estimated using a two−site exchange model (18). Between−group comparisons of AUC or single−time outcomes were performed using one−way ANOVA with Tukey’s *post hoc* tests (adjusted *p*-values). Two−group comparisons used Student’s two−tailed t−tests. A *p*-value < 0.05 was considered statistically significant. Analyses were conducted in GraphPad Prism 6.0 (GraphPad Software, CA, USA).

## 3 Results

### 3.1 ACLY inhibition reduces alanine- and glutamine-driven hyperglycemia in the db/db mice

We previously observed that ACLY overexpression increased glucose labeling in primary hepatocytes (**Supplementary Figure S2A**), while BMS-303141 suppressed glucagon-mediated glucose output (**Supplementary Figure S2B**).

**Figure 1:**
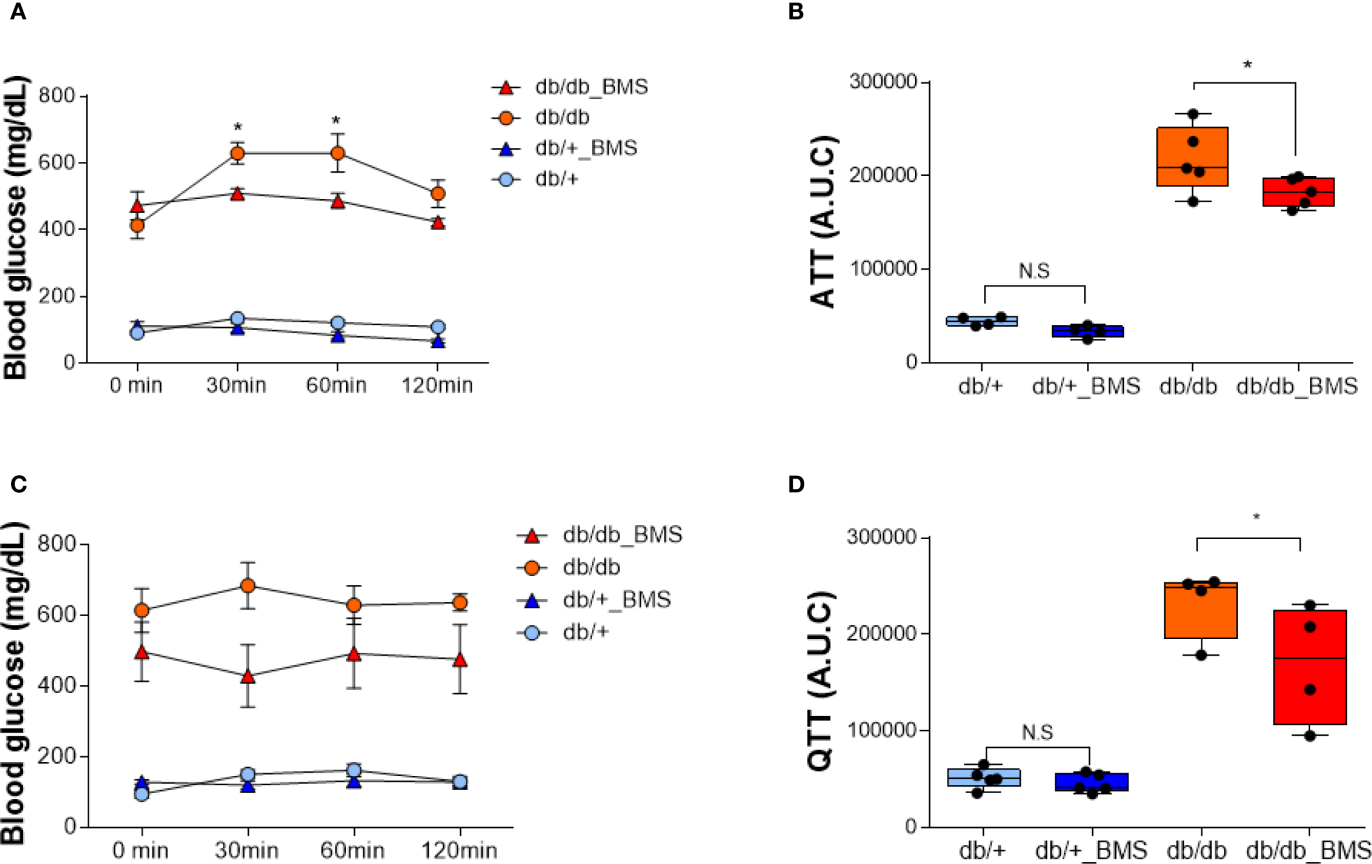
ACLY inhibition attenuates alanine- and glutamine-induced hyperglycemia in the db/db mice. **(A, C)** Blood glucose levels in db/+ and db/db mice following intraperitoneal injection of 1 g/kg L-alanine **(A)** or L-glutamine **(C)**, with or without BMS-303141 pretreatment (2.5 mg/kg, oral, 2 h prior). Data are shown as mean ± SEM (db/+ n = 4, db/db n = 5). Time course curves were analyzed by two-way repeated measures ANOVA (factors: group × time) with Tukey’s *post hoc* tests; adjusted *p* < 0.05. **(B, D)** Area under the curve (AUC) analysis of the alanine tolerance test **(B)** and glutamine tolerance test **(D)**. Group comparisons were performed by one-way ANOVA with Tukey’s *post hoc* test; adjusted *p* < 0.05; N.S., not significant. Group comparisons were performed by one-way ANOVA with Tukey’s post hoc test; *p < 0.05; N.S., not significant.

In db/db mice, a single oral dose (2.5 mg/kg) of the ACLY inhibitor BMS-303141 did not significantly reduce fasting blood glucose (**Supplementary Figure S3A, B**). However, pretreatment with BMS-303141 markedly attenuated alanine- and glutamine-induced hyperglycemia (**Figures 1A–D**), suggesting that ACLY activity contributes to hepatic glucose labeling through amino acid–mediated pathways, likely involving ALT transamination. This supports the hypothesis that ACLY plays a regulatory role in ALT-mediated gluconeogenesis in diabetic liver.

### 3.2 HP [1-^13^C]alanine flux is elevated in diabetic liver but rapidly normalized by ACLY inhibition

**Figure 2:**
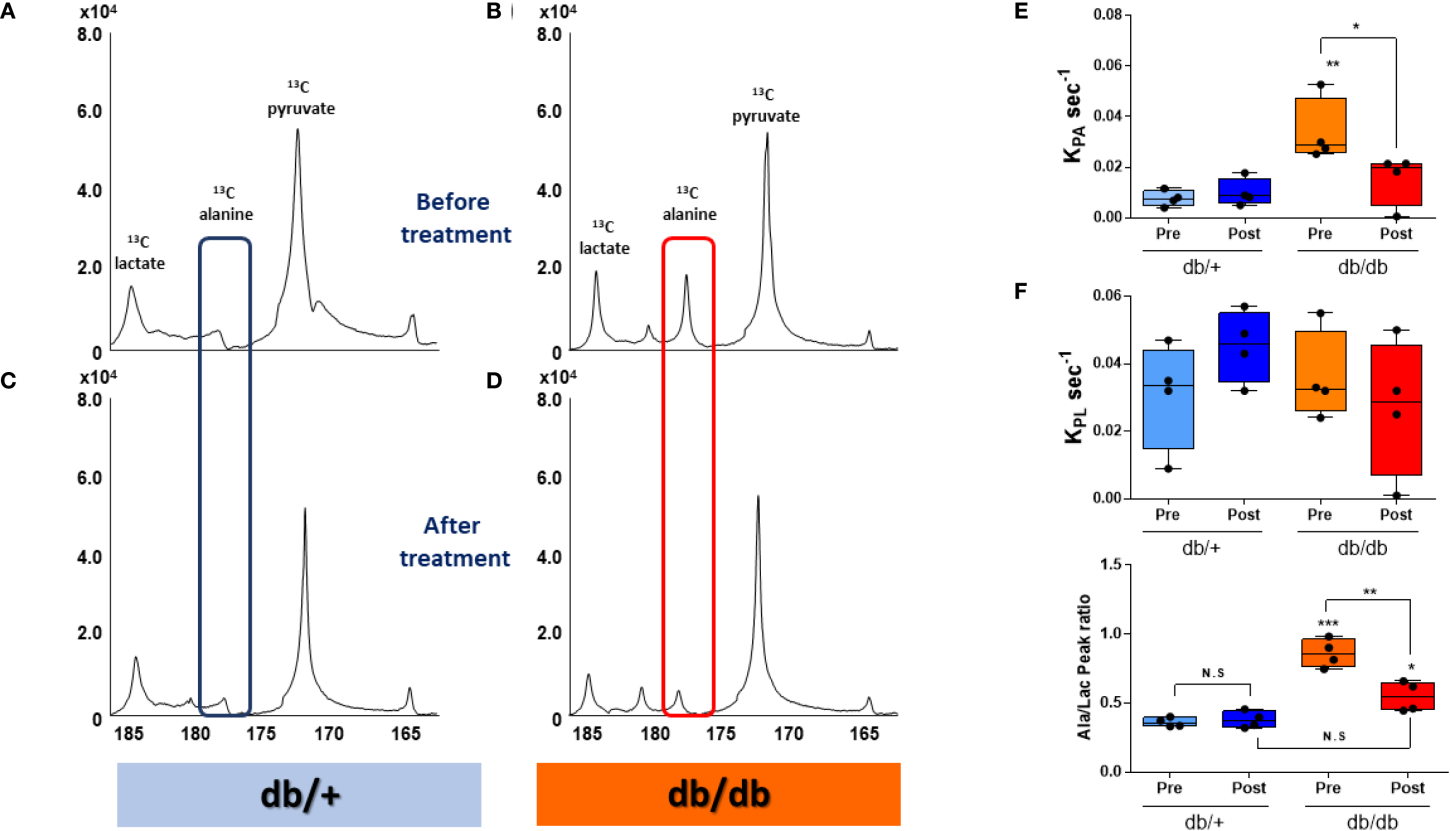
HP [1-^13^C]alanine flux is elevated in db/db livers and normalized by ACLY inhibition. **(A–D)** Representative HP [1-^13^C] MR spectra before treatment before **(A, B)** and after 2–4 h after **(C, D)** BMS-303141 (2.5 mg/kg) administration. Peaks: pyruvate (173 ppm), alanine (178.5 ppm), and lactate (185 ppm). Alanine peaks are highlighted with a blue box in db/+ mice and a red box in db/db mice. **(E)** Pyruvate-to-alanine rate constant (*KpA).* **(F)** pyruvate-to-lactate rate constant *(KpL)* **(G)** Alanine/lactate peak signal ratio. Group comparisons were performed by one-way ANOVA with Tukey’s *post hoc* test: adjusted *p < 0.05, **p < 0.01, ***p < 0.001; N.S., not significant.

HP [1-^13^C]pyruvate MRS revealed a markedly elevated [1-^13^C]alanine signal in the liver of db/db mice following a 20-hour fast (**Figure 2B**). Remarkably, this signal returned to near-control levels within 2–4 hours after administration of BMS-303141 (**Figure 2D**). In contrast, the same treatment did not significantly alter the alanine signal in db/+ control mice (**Figures 2A, C**). HP [1-^13^C]lactate levels remained unchanged across groups, indicating a specific alteration in alanine metabolism.

Correspondingly, the calculated *KpA* was significantly elevated in db/db mice and normalized upon ACLY inhibition (**Figure 2E**). *KpL* showed no significant change (**Figure 2F**). The alanine-to-lactate signal ratio, which was highest in db/db mice, was partially restored by BMS-303141 treatment (**Figure 2G**). Given that [1-^13^C]alanine reflects ALT-mediated transamination of pyruvate, the observed changes provide dynamic evidence consistent with altered pyruvate-alanine exchange.

### 3.3 Elevated ALT and AST enzyme activity assay results are consistent with the increased alanine flux observed in db/db livers

**Figure 3:**
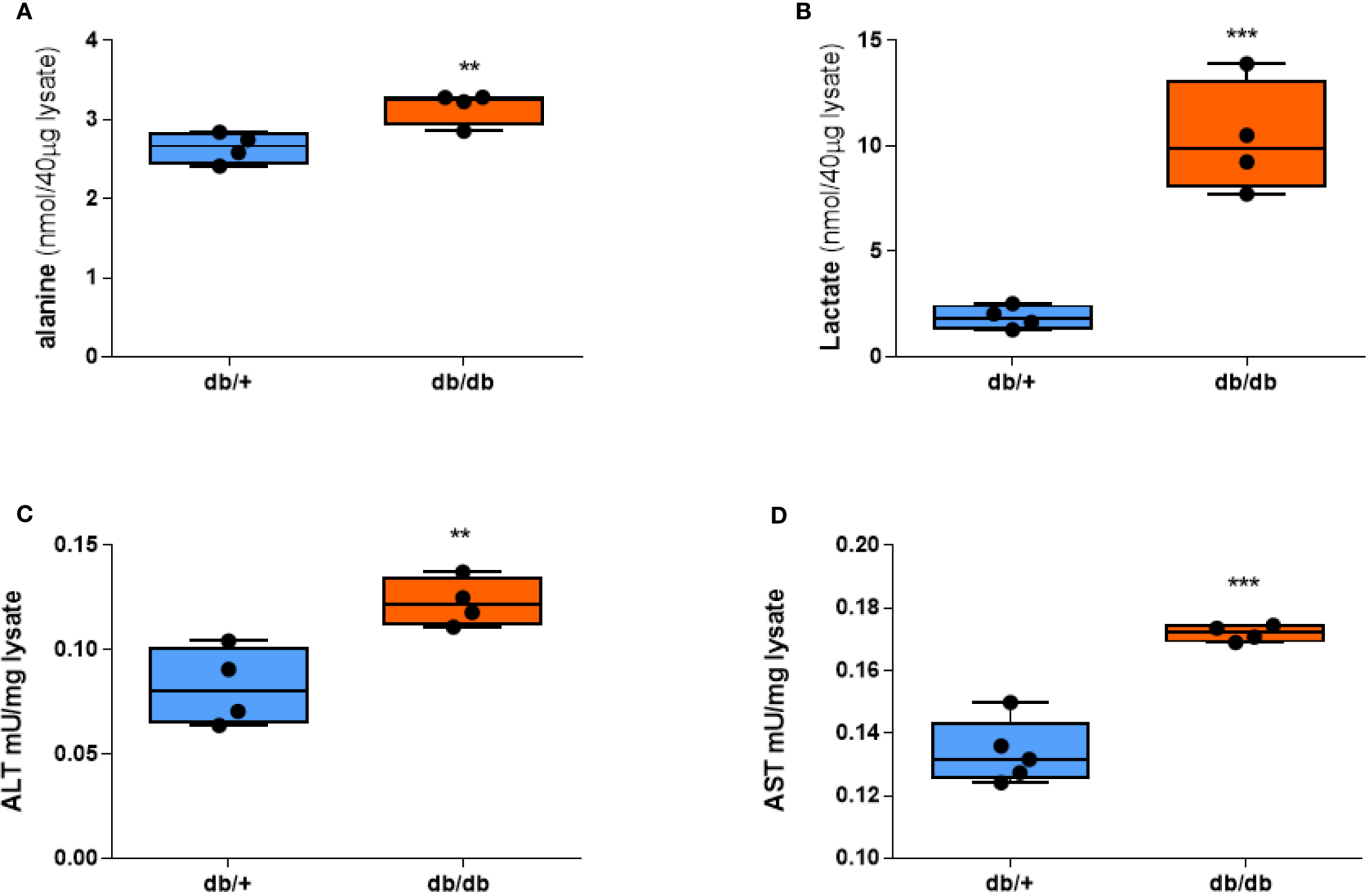
Alanine, lactate, and aminotransferase activities are elevated in db/db mice. **(A)** Alanine concentration. **(B)** Lactate concentration. **(C)** ALT activity. **(D)** AST activity in liver tissue after a 20-hour fast. Group comparisons were performed by Student’s two-tailed t-test; **p < 0.01, ***p < 0.001.

Since the signal intensity of HP [1-^13^C]alanine and HP [1-^13^C]lactate reflects the amount of lactate and alanine in tissue (18), we measured the concentrations of lactate and alanine in the livers of db/+ and db/db mice. db/db livers showed significantly higher alanine content compared to db/+ (**Figure 3A**). Lactate levels in the db/db liver were also higher than db/+ (**Figure 3B**), even though HP [1-^13^C]lactate flux did not distinguish between them. ALT enzyme activity, as determined by a colorimetric assay, was also significantly increased (**Figure 3C**), as was AST enzyme activity (**Figure 3D**), suggesting a broader upregulation of aminotransferase pathways under diabetic conditions. These ex vivo assay results support the notion that enhanced ALT function may contribute to the increased hepatic alanine flux observed in diabetic mice. However, the mRNA expression of aminotransferase genes, including ALT1, ALT2, AST1, and AST2, did not differ between groups (**Supplementary Figure S4**).

### 3.4 ACLY is functionally activated in db/db mice and correlates with increased alanine flux

**Figure 4:**
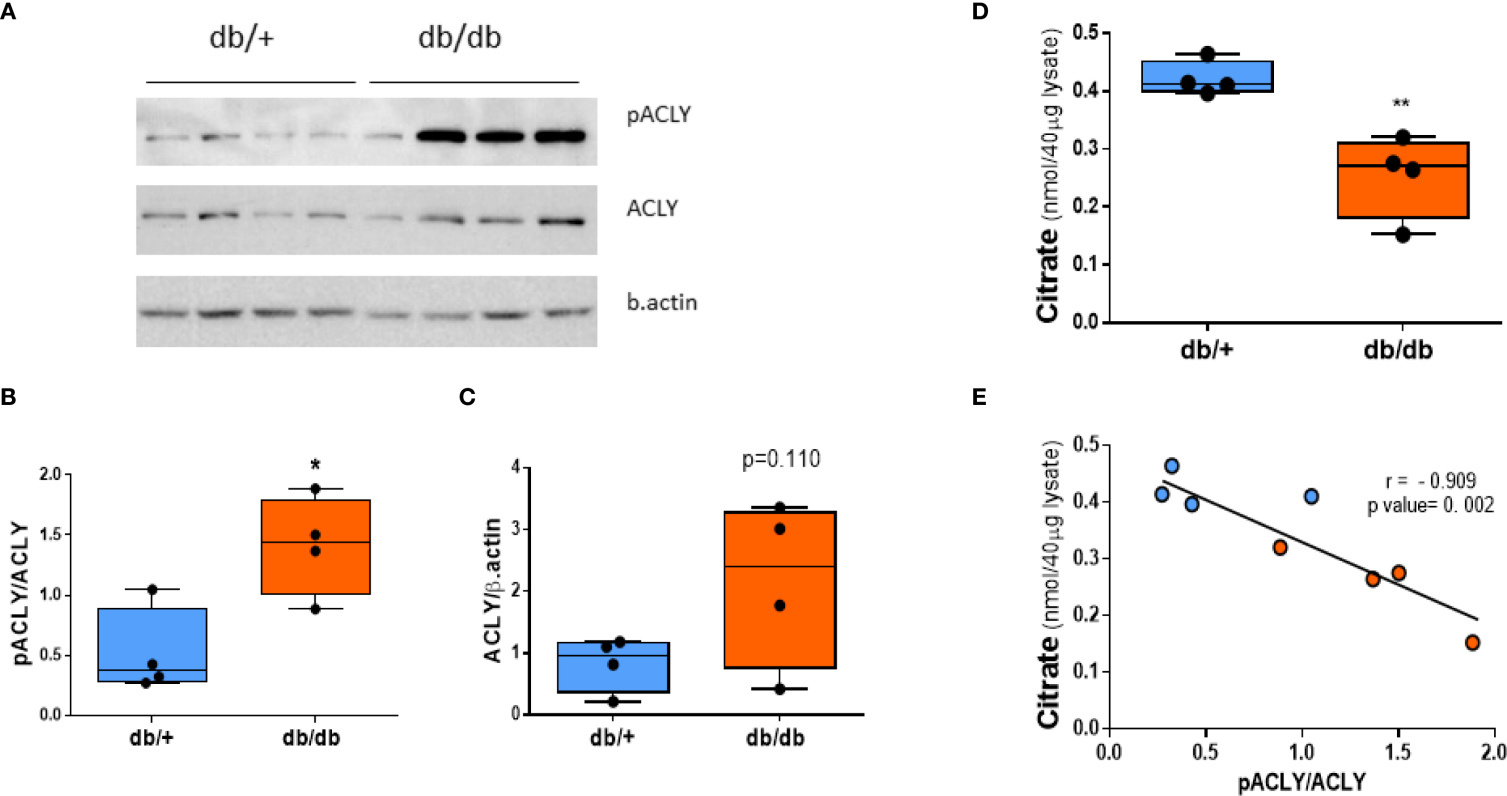
ACLY is phosphorylated (activated) in db/db liver. **(A)** Western blot of phospho-ACLY (Ser455), total ACLY, and β-actin. **(B)** Densitometric analysis of pACLY/ACLY ratio. **(C)** Total ACLY expression. **(D)** Citrate levels in liver tissue. **(E)** Correlation between citrate concentration and pACLY/ACLY ratio. Blue circles: db/+ mice; orange circles: db/db mice. Comparisons between groups were assessed by Student’s two-tailed t-test; *p < 0.05, **p < 0.01.

Western blot analysis showed comparable total ACLY protein levels between db/+ and db/db livers. However, phosphorylated ACLY at Ser455—the active form—was significantly increased in db/db mice (**Figures 4A, B**). Citrate concentrations were reduced in db/db livers (**Figure 4D**) and inversely correlated with the pACLY/ACLY ratio (**Figure 4E**), consistent with enhanced ACLY activity and citrate cleavage.

**Figure 5:**
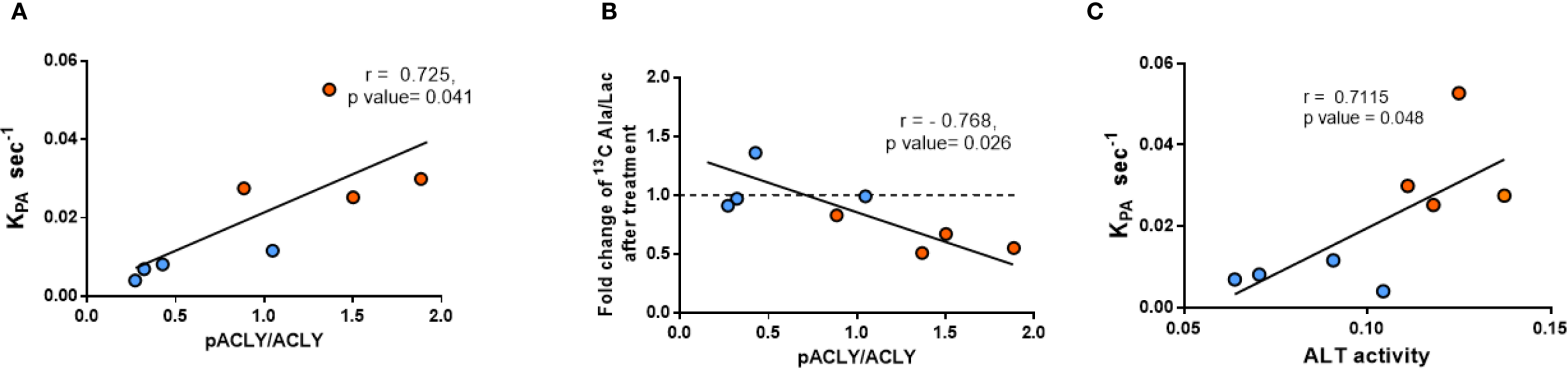
Correlation between ACLY phosphorylation, ALT activity, and HP [1-^13^C]alanine labeling. **(A)** Correlation between pACLY/ACLY ratio and *KpA*. **(B)** Correlation between pACLY/ACLY ratio and fold change in alanine/lactate ratio after BMS-303141. **(C)** Correlation between hepatic ALT activity and *KpA*. Blue circles: db/+ mice; orange circles: db/db mice. Statistical significance determined by Pearson’s correlation (r, p values shown on plots).

A strong positive correlation was observed between the pACLY/ACLY ratio and the *KpA* value (**Figure 5A**) supporting a functional link between ACLY activity and ALT-mediated alanine flux. Furthermore, mice with higher pACLY/ACLY ratios exhibited a greater fold reduction in alanine/lactate ratio following BMS-303141 treatment (**Figure 5B**), indicating that ACLY inhibition suppresses ALT-mediated transamination *in vivo*. Consistent with this, hepatic ALT1 activity also positively correlated with the *KpA* (**Figure 5C**), further reinforcing the association between ALT activity and alanine flux. While the correlation between ALT1 activity and *KpA* does not imply causality, it supports a functional relationship between ALT pathway activity and hepatic alanine flux. However, the mRNA expression of ACLY and its upstream citrate carrier (CIC, Slc25A1) showed no significant difference between db/+ and db/db mice (**Supplementary Figure 4**), indicating that ACLY hyperactivation is likely due to enhanced metabolic flux or signaling-driven enzyme activation rather than transcriptional changes.

## 4 Discussion

Since ACLY supplies cytosolic OAA and acetyl-CoA, it plays a central role in supporting both gluconeogenesis and fatty acid synthesis—two key metabolic pathways that are paradoxically elevated in T2DM and NAFLD. Prompted by our observation of markedly elevated alanine flux in the diabetic liver using hyperpolarized [1-^13^C]pyruvate MRS (HP-MRS), we investigated how ACLY contributes to amino acid–driven gluconeogenesis under diabetic conditions using db/db mice. We found that pyruvate-to-alanine flux was markedly increased in the diabetic liver, and this elevation was rapidly normalized following ACLY inhibition. Consistently, a similar increase in hepatic HP [1-^13^C]alanine was reported in Zucker diabetic fatty rats, another leptin receptor–deficient model, indicating that enhanced alanine flux is a shared metabolic feature of leptin resistance (19). These metabolic changes occurred without transcriptional upregulation of ALT1, ALT2, or ACLY itself, indicating that the effects are likely driven by enzyme activation or altered metabolic routing. Consistent with this, we observed a significant increase in the phosphorylated, catalytically active form of ACLY (pACLY) in db/db livers, without a corresponding change in total protein expression. Together, these findings suggest that ACLY activation under diabetic conditions facilitates ALT-mediated gluconeogenesis by enhancing aminotransferase pathway activity through metabolic control rather than gene expression changes.

Several studies have demonstrated that targeting ACLY ameliorates hepatic steatosis and hyperglycemia, yet its role in hepatic gluconeogenesis has remained largely unexplored (10, 11). A recent study reported that bempedoic acid, an FDA-approved ACLY inhibitor, alleviates NASH and improves glycemic control primarily by suppressing lipogenesis and inflammation (20). Their findings also support a potential link between ACLY inhibition and reduced gluconeogenic activity. Building upon these insights, our study adds mechanistic clarity by demonstrating that ACLY promotes amino acid–driven gluconeogenesis through enhanced ALT-mediated alanine flux, independent of gene expression changes. Using hyperpolarized [1-^13^C]pyruvate MRS (HP-MRS), we demonstrate that ACLY activity is closely associated with real-time hepatic alanine labeling, underscoring its broader role in coordinating amino acid metabolism under diabetic conditions.

**Figure 6:**
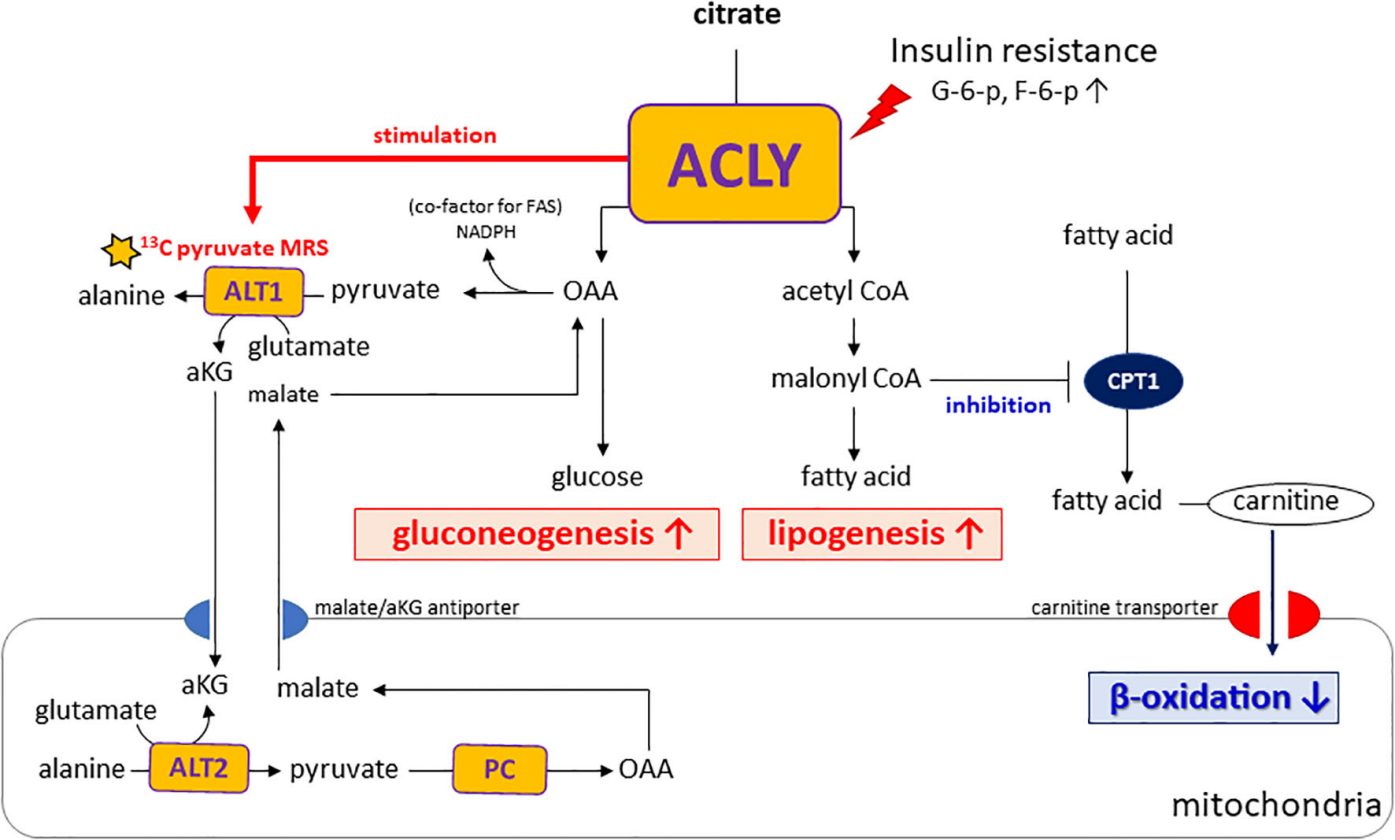
Proposed model of ACLY in the metabolic dysregulation of NAFLD and T2DM. Insulin resistance increases glucose-6-phosphate (G6P) and fructose-6-phosphate (F6P), which activate ACLY. Activated ACLY generates cytosolic oxaloacetate (OAA) and acetyl-CoA. OAA supports gluconeogenesis, while acetyl-CoA is converted to malonyl-CoA, promoting lipogenesis and inhibiting β-oxidation via CPT1 suppression. ACLY activation also enhances ALT1/ALT2–mediated alanine transamination and αKG–malate cycling, thereby linking amino acid metabolism to gluconeogenic flux. Together, these pathways explain the simultaneous increase in gluconeogenesis and lipogenesis observed in NAFLD and T2DM.

Importantly, insulin resistance may contribute to the functional activation of ACLY, as elevated levels of glucose-6-phosphate and fructose-6-phosphate—common in hyperglycemic states—are known allosteric activators of ACLY (21). This activation likely drives the concurrent labeling of acetyl-CoA and OAA from citrate, thereby supporting both lipogenesis and gluconeogenesis. In addition, alanine generated in the cytosol via ALT1 can be transported into mitochondria and converted back to pyruvate through ALT2. This mitochondrial pyruvate is then carboxylated by pyruvate carboxylase (PC) to OAA, which is subsequently reduced to malate and exported to the cytosol via the malate/αKG shuttle, thereby contributing to gluconeogenesis. As illustrated in our proposed metabolic model (**Figure 6**), ACLY may thus serve as a central metabolic node that integrates carbohydrate overload with increased hepatic glucose output, providing a biochemical basis for the metabolic paradox observed in NAFLD and T2DM.

A notable aspect of our study is the use of HP [1-^13^C]pyruvate MRS to monitor hepatic metabolic fluxes *in vivo*. This technique enabled us to capture the rapid shift in alanine labeling following ACLY inhibition, offering functional insight beyond static metabolite or transcript levels. Elevated alanine labeling in db/db mice likely reflects increased ALT1 activity, which catalyzes the transamination of pyruvate to alanine in the cytosol. Importantly, this reaction generates α-ketoglutarate (αKG). αKG participates in the αKG/malate antiport system that supports gluconeogenic flux by enabling malate export from mitochondria. Because cytosolic ACLY activity depends on citrate export via the CIC, which is coupled to αKG/malate exchange through OGC (oxoglutarate carrier, Slc25A11), ACLY activation may enhance this transamination cycle by increasing cytosolic αKG availability. As ALT transamination requires glutamate as the nitrogen donor, enhanced αKG/glutamate cycling provides a plausible intermediary mechanism linking ACLY activation to increased alanine labeling and gluconeogenesis under insulin-resistant conditions (**Supplementary Figure S5**). These observations highlight how enhanced alanine flux may not only reflect amino acid metabolism but also serve as an indicator of broader rewiring of hepatic energy pathways in diabetic states.

Elevated ALT activity in T2DM and obesity has been associated with increased alanine release from skeletal muscle, thereby contributing to hyperglycemia (22, 23). These findings highlight the potential of ALT as a metabolic mediator and diagnostic marker in metabolic diseases such as NAFLD and T2DM. However, several studies have shown that serum ALT levels do not reliably reflect hepatic enzymatic activity or liver pathology (24). In line with this, our study found no significant difference in serum ALT levels despite marked changes in hepatic alanine metabolism (**Supplementary Figure S6**). This underscores the value of HP [1-^13^C]pyruvate MRS, which directly captures hepatic transamination activity *in vivo* and may serve as a more accurate non-invasive tool for monitoring metabolic dysfunction and treatment response.

In interpreting HP [1-^13^C]pyruvate MRS data, it is important to recognize that the detected [1-^13^C]alanine and [1-^13^C]lactate signals reflect not only intracellular pool sizes (18) but also the relative activities of key enzymes, particularly ALT and LDH. While lactate labeling typically dominates in many tissues due to high LDH activity, the liver presents a distinct metabolic environment where ALT activity can be substantially higher. This preferential routing of pyruvate toward alanine is supported by prior studies using perfused liver slices, which demonstrated that hepatic ALT activity is approximately 2.3 times higher than LDH activity, underscoring the hepatic propensity for alanine labeling (25).

Despite these insights, our study has several limitations. First, while the db/db mouse is a well-established model of T2DM, its pathophysiology may not fully recapitulate human NAFLD. Second, although we observed altered alanine flux with ACLY inhibition, we did not directly assess post-translational modifications or subcellular localization of ALT1 versus ALT2, which could clarify the mechanism of enhanced flux. Third, we did not quantify intrahepatic levels of BMS-303141 or assess longer time points beyond 4 hours, limiting our understanding of the full temporal dynamics of ACLY inhibition. Fourth, although BMS-303141 is widely used as an ACLY inhibitor, potential off-target effects cannot be excluded, and the long-term metabolic consequences of ACLY inhibition require further evaluation. Finally, the colorimetric alanine/lactate and ALT/AST assays reflect apparent enzymatic activity in tissue lysates under ex vivo conditions rather than direct *in vivo* activity. We did not perform independent specificity validation, so matrix effects or cross-reactivity cannot be ruled out. Accordingly, we regard these ex vivo assays as complementary to, rather than causal proof of, the *in vivo* HP [1-^13^C]pyruvate MRS findings.

In conclusion, our findings reveal a previously underappreciated role for ACLY in regulating ALT-mediated gluconeogenesis in the diabetic liver. By leveraging hyperpolarized [1-^13^C]pyruvate MRS (HP-MRS), we demonstrate that ACLY inhibition not only reduces alanine-driven glucose labeling but also modulates hepatic energy metabolism *in vivo*. These results highlight ACLY as a potential dual-action therapeutic target in NAFLD and T2DM, and establish HP ^13^C MRS as a powerful translational tool for evaluating dynamic hepatic metabolic flux and therapeutic responses in metabolic disease. Future studies assessing the subcellular localization and post-translational modifications of ALT isoforms, as well as applying this imaging strategy in diet-induced models or human samples, will further clarify the translational potential of our findings.

## Data Availability

The original contributions presented in the study are included in the article/**Supplementary Material**. Further inquiries can be directed to the corresponding author.
