# Supplementary material for: ACLY facilitates alanine flux in the livers of db/db mice: a hyperpolarized [1-13C]pyruvate MRS study
Source: Front Endocrinol (Lausanne). 2025 Oct 27;16:1663958. doi: 10.3389/fendo.2025.1663958 (PMC12597765; doi:10.3389/fendo.2025.1663958)
Supplement: Supplementary file 1 [file DataSheet1.docx]

**
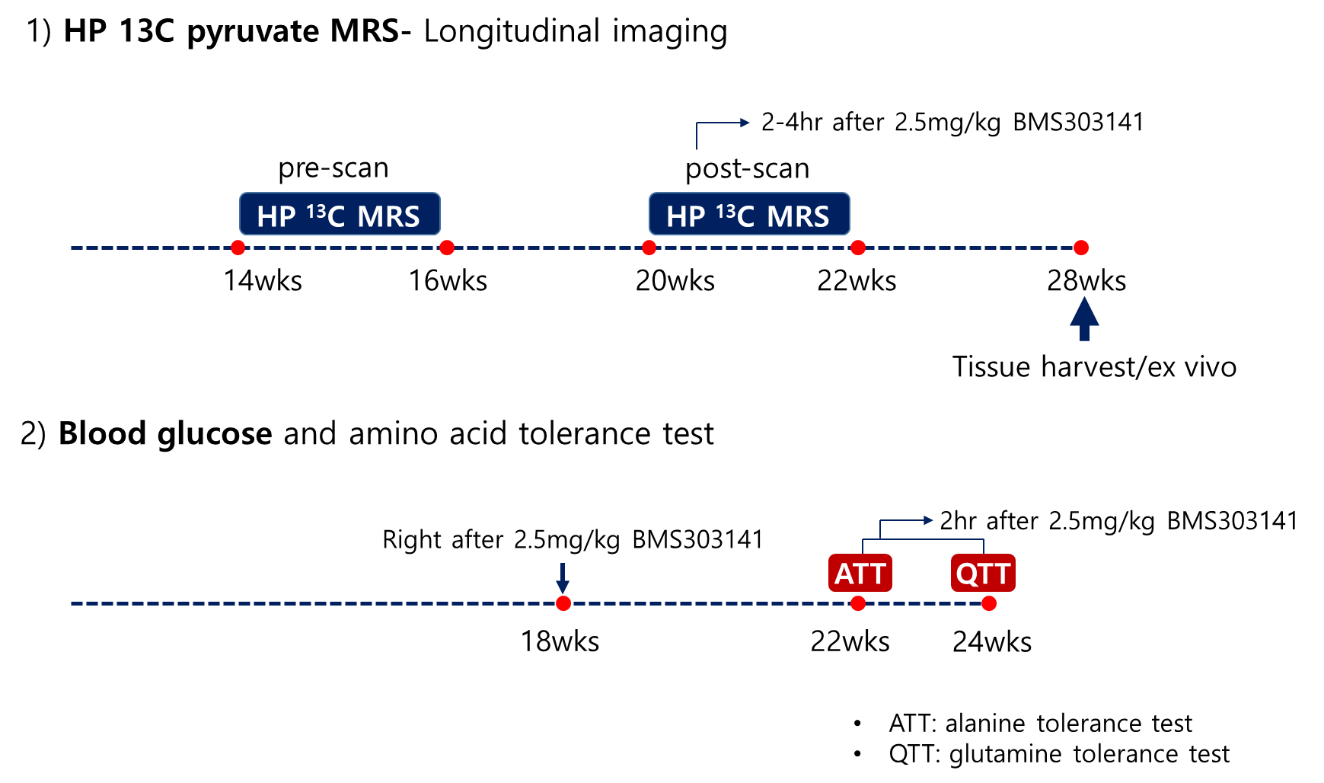
**

**Supplementary Figure S1. Timeline of experimental procedures.**

Pre‑scan HP [1‑^13^C]pyruvate MRS was performed at 14–16 weeks after overnight fasting. Post‑scan HP [1‑^13^C]pyruvate MRS was performed at 20–22 weeks, 2–4 h after oral BMS‑303141 (2.5 mg/kg).

Alanine and glutamine tolerance tests were conducted at 18 weeks with or without 2‑h BMS pretreatment. Tissues for ex vivo analyses were harvested at 28 weeks from the imaged cohort after overnight fasting. Red dots indicate experimental time points.

**
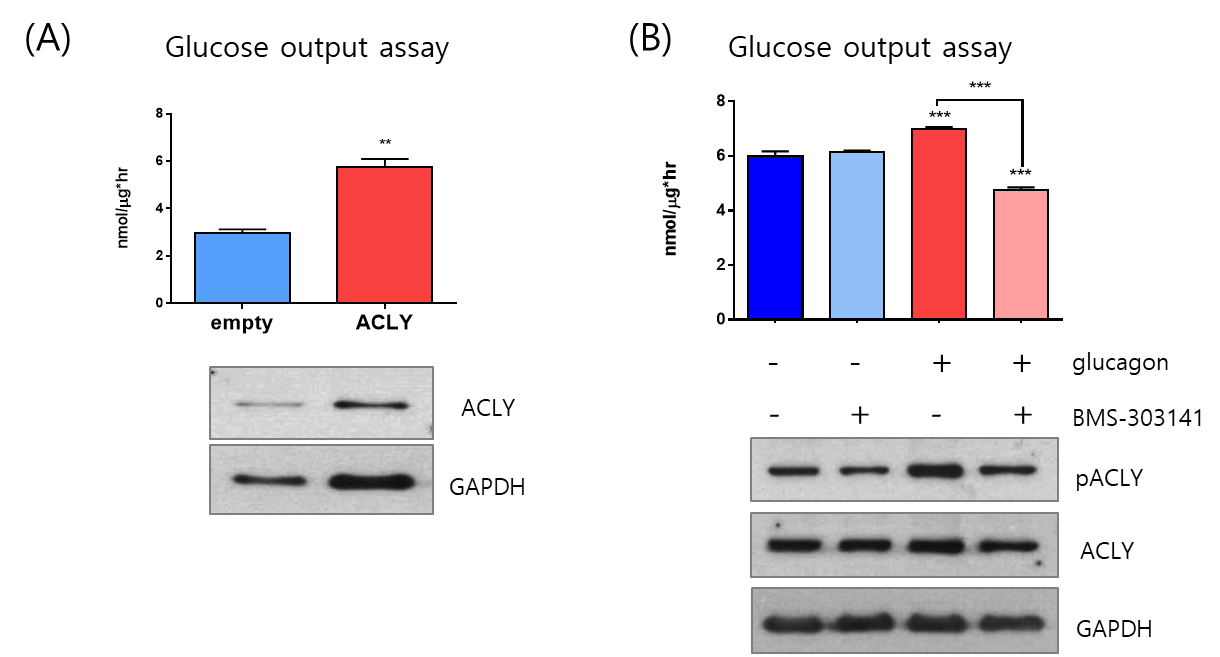
**

**Supplementary Figure S2. ACLY inhibitor reduces glucose output in primary hepatocytes.**

(A) ACLY overexpression increases glucose production in mouse primary hepatocytes.

(B) ACLY inhibition by BMS-303141 reduces glucagon-mediated glucose output.

**
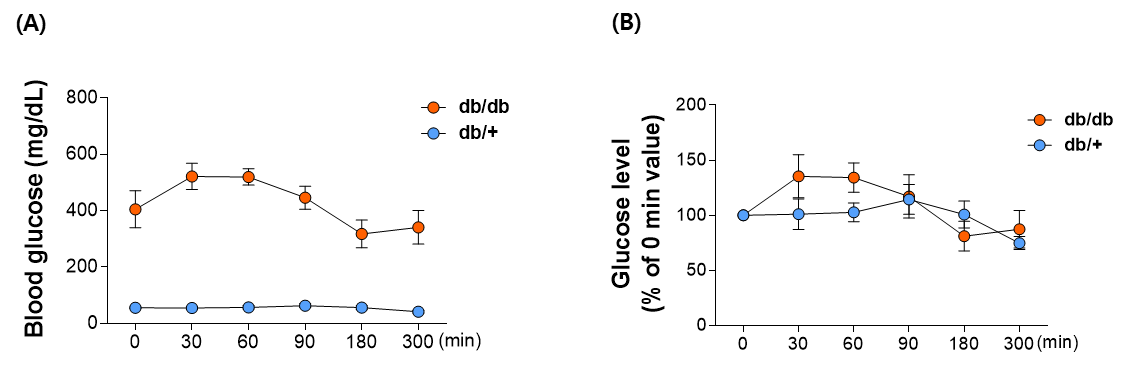
**

**Supplementary Figure S3. BMS-303141 does not significantly reduce fasting blood glucose in db/db mice.**

(A) After a 20-hour fast, blood glucose levels were measured in 18-week-old db/+ and db/db mice at 0, 30, 60, 90, 180, and 300 minutes after oral administration of 2.5 mg/kg BMS-303141. (B) Change in glucose levels from the initial point during the administration of 2.5 mg/kg BMS-303141.

**
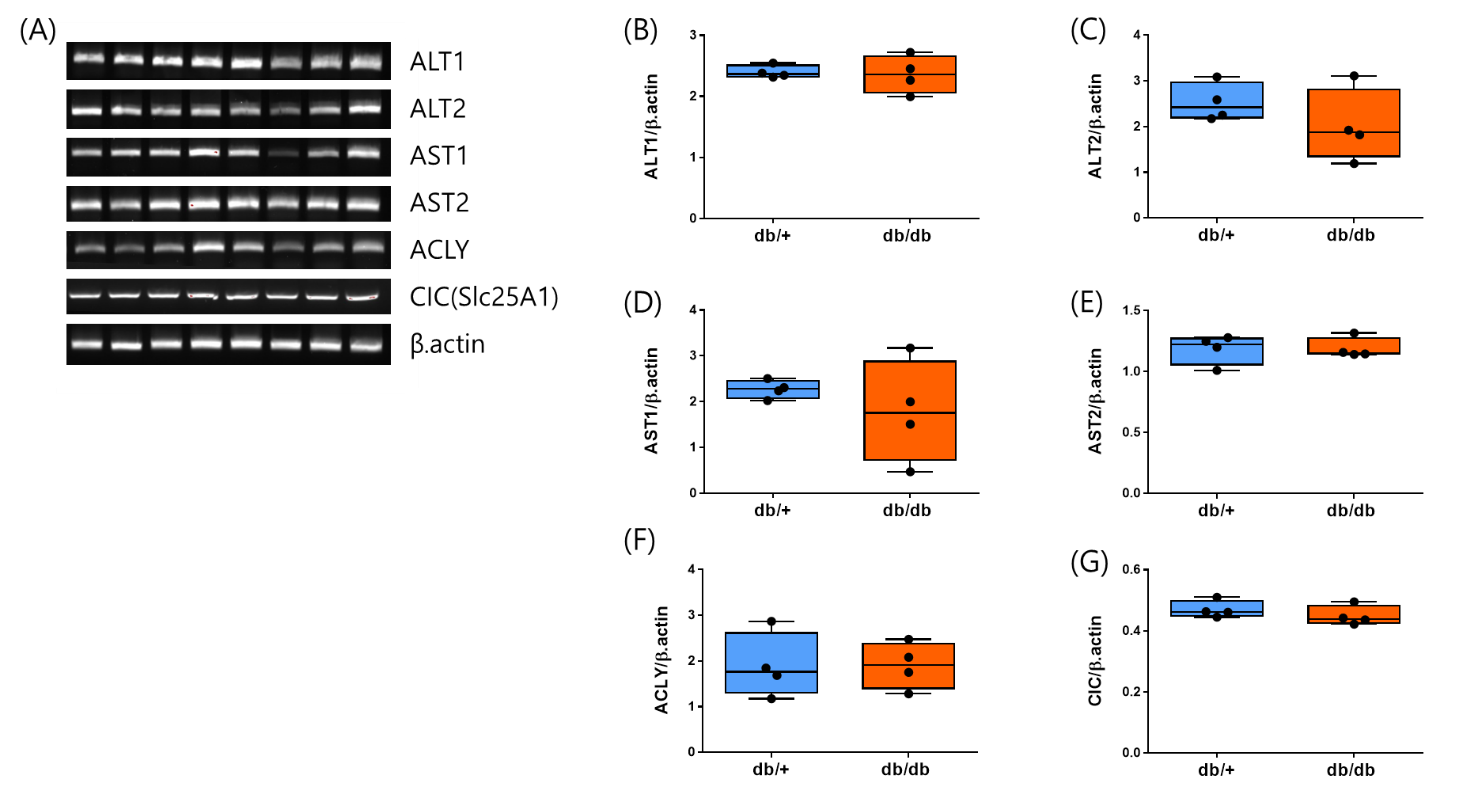
**

**Supplementary Figure S4. Gene expression analysis of aminotransferases and citrate metabolism enzymes in db/+ and db/db mouse livers.**

(A) Representative RT-PCR gel images showing mRNA expression levels of ALT1, ALT2, AST1, AST2, ACLY, and CIC (Slc25a1) in liver tissue.

(B–G) Quantification of mRNA expression levels by densitometry analysis, normalized to β-actin.

(B) ALT1, (C) ALT2, (D) AST1, (E) AST2, (F) ACLY, and (G) CIC mRNA levels showed no significant differences between db/+ and db/db mice.

Data are presented as mean ± SEM (n = 4 per group).


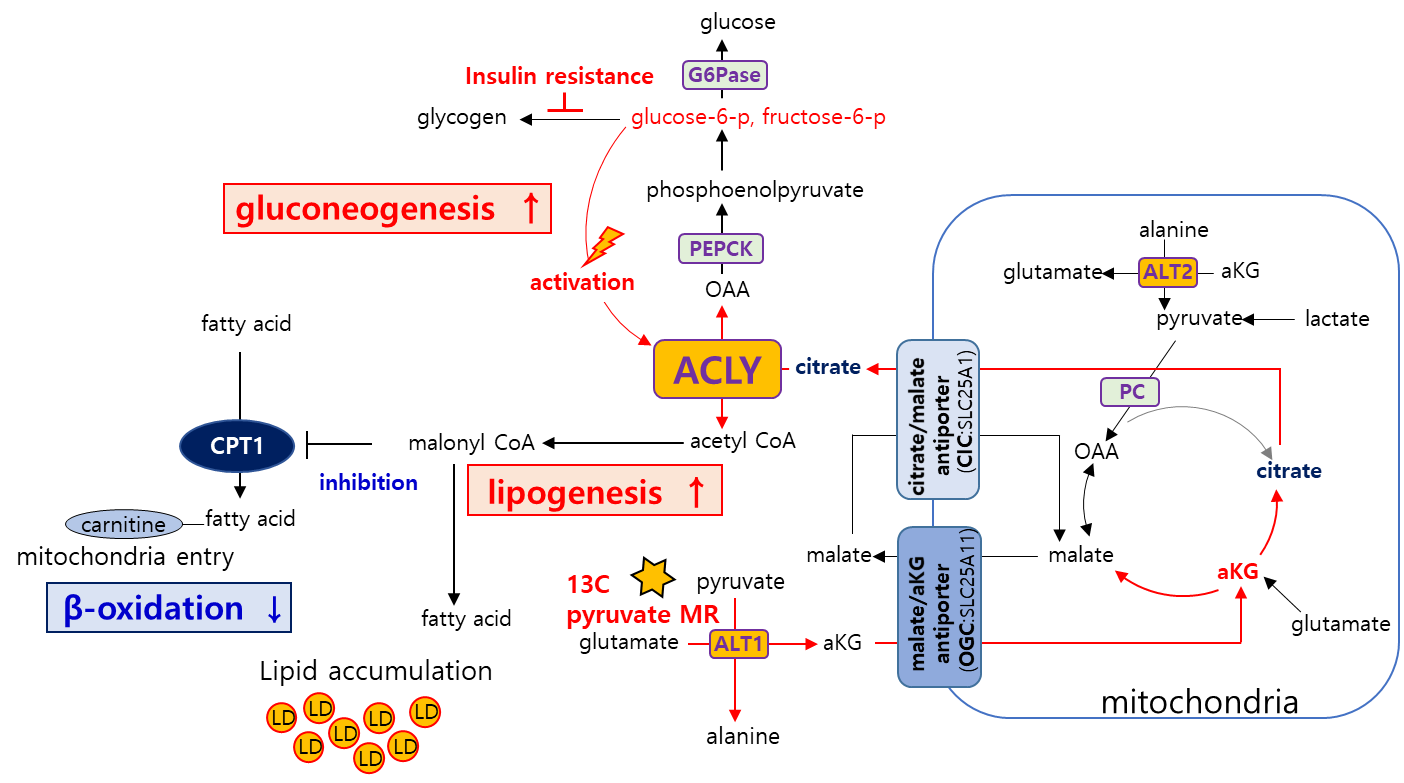


**Supplementary Figure S5. Proposed role of ACLY in metabolic reprogramming under insulin resistance.**

Schematic representation of ACLY-mediated regulation of alanine metabolism and lipid accumulation under insulin-resistant conditions.

In hyperglycemia and insulin resistance, elevated glucose-6-phosphate and fructose-6-phosphate activate ACLY. Citrate exported from mitochondria via the citrate carrier (CIC, SLC25A1) and coupled malate/α-ketoglutarate exchange through OGC (SLC25A11) provides substrates for ACLY activity and increases cytosolic αKG availability. This shuttle promotes ALT1/ALT2-dependent alanine flux, thereby enhancing gluconeogenesis. At the same time, ACLY generates acetyl-CoA, which drives lipogenesis and produces malonyl-CoA that inhibits CPT1, suppressing β-oxidation. These processes collectively increase hepatic glucose output and lipid droplet accumulation, linking ACLY activation to the paradoxical coexistence of enhanced gluconeogenesis and lipid deposition in NAFLD and T2DM.

**Supplementary Figure S6. Serum ALT levels showed no difference between groups.**

(A) plasma ALT were measured in control (db/+) and db/db mice. No significant difference was observed between the two groups.
